# Facile synthesis and antiproliferative activity of new 3-cyanopyridines

**DOI:** 10.1186/s13065-019-0652-1

**Published:** 2019-12-28

**Authors:** Hassan M. Abdel-aziz, Sobhi M. Gomha, Abdelaziz A. El-Sayed, Yahia Nasser Mabkhot, Abdulrhman Alsayari, Abdullatif Bin Muhsinah

**Affiliations:** 1 Chemistry Department, Faculty of Science, University of Bani Suef, Bani Suef, Egypt; 2 Chemistry Department, Faculty of Science, University of Cairo, Giza, 12613 Egypt; 3 Chemistry Department, Faculty of Science, Islamic University in Almadinah Almonawara, Almadinah Almonawara, 42351 Saudi Arabia; 4 Zoology Department, Faculty of Science, Zagazig University, Zagazig, Egypt; 5 Department of Pharmaceutical Chemistry, College of Pharmacy, King Khalid University, Abha, Saudi Arabia; 6 Department of Pharmacognosy, College of Pharmacy, King Khalid University, Abha, Saudi Arabia

**Keywords:** 2-Cyanoacetohydrazide, Cyclization, 3-Pyridinecarbonitrles, 3-Quinolinecarbonitriles, Antitumor activity

## Abstract

**Background:**

Pyridines have been reported to possess various pharmacological activities.

**Results:**

Sodium 3-oxo-3-(2-oxo-2*H*-chromen-3-yl)prop-1-en-1-olate (**2**) and sodium 3-oxo-3-(3-oxo-3*H*-benzo[f]chromen-2-yl)prop-1-en-1-olate (**7**) were prepared and reacted with 2-cyano-*N*’-(1-aryl(heteryl)ethylidene)acetohydrazides **3a–d** to produce 2-oxo-1,2-dihydropyridine-3-carbonitrile derivatives **5a–d** and **9a–d**, respectively, in good yields. Also, **3a–d** reacted with sodium (2-oxocyclopentylidene)methanolate (**11a)** or sodium (2-oxocyclohexylidene) methanolate **(11b**) to yield 2-oxo-tetrahydro-1*H*-cyclopenta[b]pyridine-3-carbonitriles **13a–d** and 2-oxo-hexahydroquinoline-3-carbonitriles **13e–h**, respectively. The mechanisms that account for the formation of the products are discussed. Additionally, the structures of all the newly synthesized products are confirmed, based on elemental analysis and spectral data. Several of the newly synthesized compounds are evaluated for their antitumor activity against HEPG2 and their structure activity relationship (SAR) was studied.

**Conclusions:**

The results revealed that the pyridine derivatives **5c** and **5d** (IC_50_ = 1.46, 7.08 µM, respectively) have promising antitumor activity against liver carcinoma cell line (HEPG2), compared to the reference drug, doxorubicin. 
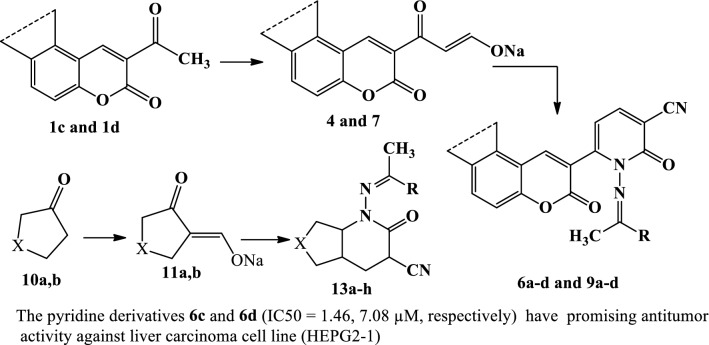

## Introduction

The pyridine core is a key constituent in a scope of bioactive compounds which occur artificially and naturally. It has been appeared to have a wide scope of biological applications [1–3]. Among these, substituted cyanopyridines were found to have antihypertensive [4], antipyretic, anti-inflammatory and analgesic properties [5]; cardiotonic [6], antimicrobial [7], and anticancer activities [8, 9]. Among the successful examples as drug candidates possessing the pyridine core are streptonigrone, lavendamycin and streptonigrin, which are depicted in the literature as anticancer agents. Some pyridine derivatives were contemplated for their topoisomerase inhibitory action and cytotoxicity against a few human malignant growth cell lines, thus marking them as novel anticancer agents [10]. Accordingly, it has been accounted those different pyridine derivatives, as bioisosteres of α-terthiophene (protein kinase C inhibitor) [11], have significant topoisomerase I and II inhibitory activity and cytotoxicity against many human cancer cell lines [12–15].

Early reports on the ability of α-terpyridine to form a metal complex [16] and to bind with DNA/RNA [17] have been the reason for the investigation of pyridine derivatives as antitumor agents. In light of the above discoveries and in continuation of our endeavors to synthesize new antitumor compounds [18–27], the aim of this report is to synthesize a new series of 3-pyridinecarbonitriles, which are anticipated to be active as antitumor agents.

## Results and discussion

**Scheme 1:**
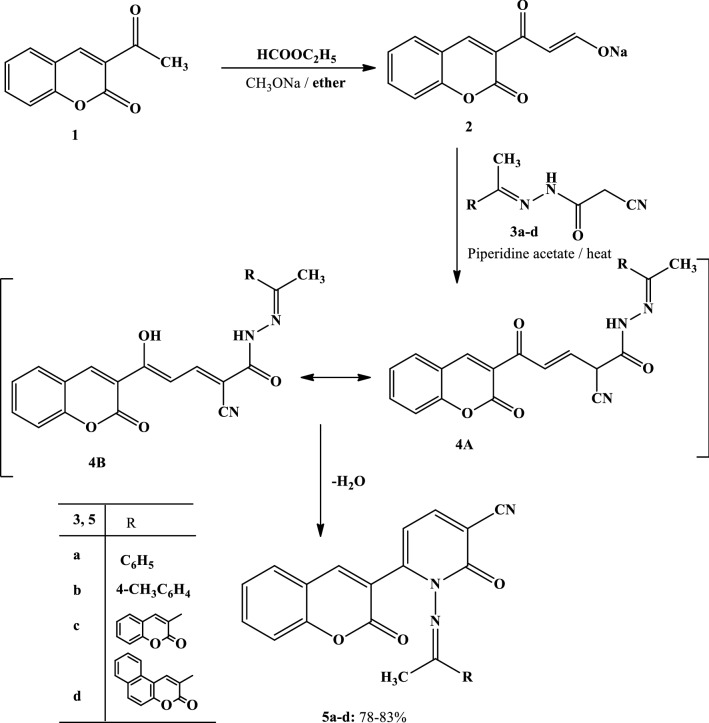
Synthesis of pyridine-3-carbonitriles **5a–d**

**Scheme 2:**
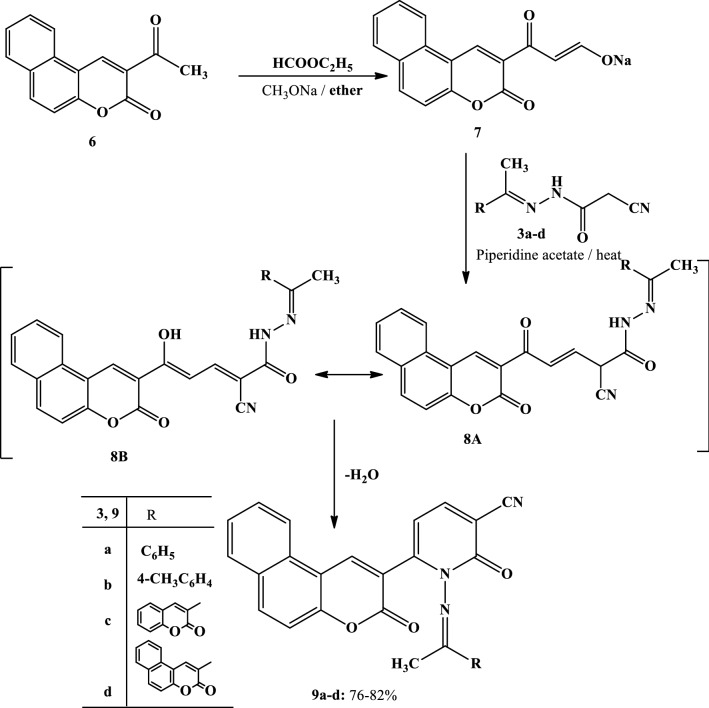
Synthesis of pyridine-3-carbonitriles **9a–d**

**Scheme 3:**
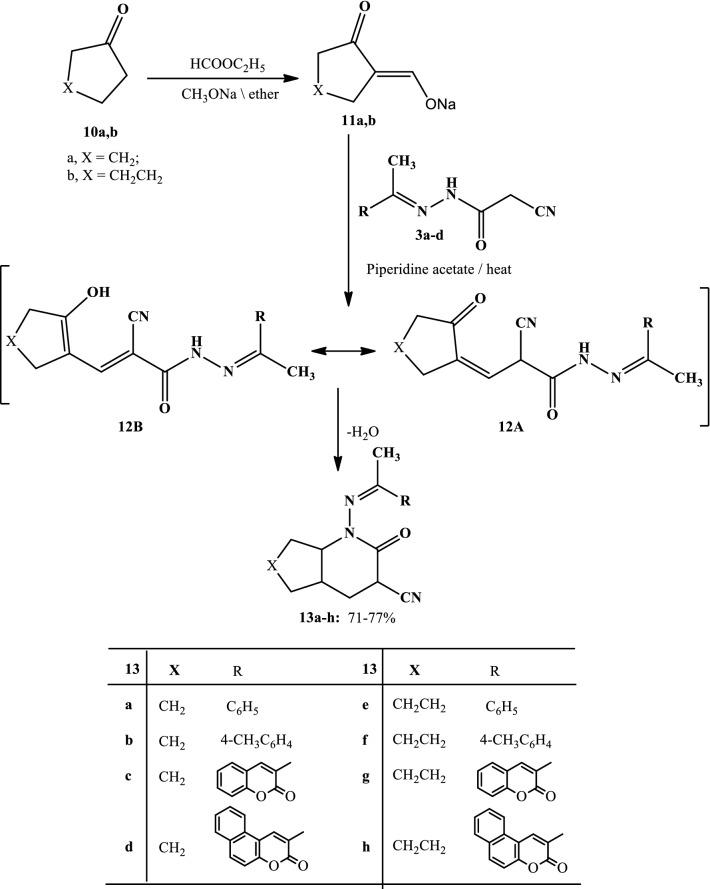
Synthesis of pyridine-3-carbonitriles **13a–h**

The synthetic strategies adopted for the synthesis of the intermediates and target compounds are depicted in Schemes 1, 2 and 3. In Schemes 1 and 2, sodium 3-oxo-3-(2-oxo-2*H*-chromen-3-yl)prop-1-en-1-olate (**2**) and sodium 3-oxo-3-(3-oxo-3*H*-benzo[f]chromen-2-yl)prop-1-en-1-olate (**7**) were prepared from a reaction of the respective 2-acetyl-3*H*-benzo[f]chromen-3-one **(1)** or 2-acetyl-3*H*-benzo[f]chromen-3-one (**6**) with ethyl formate in dry ether containing sodium methoxide, according to reported methods [28]. The structures of **2** and **7** were confirmed by chemical transformations.

The treatment of sodium salt **2 or 7** with the appropriate 2-cyano-*N*’-(1-aryl(heteryl) ethylidene)acetohydrazides **3a–d** [29–31] in acetic acid containing piperidine acetate afforded products **5a–d** and **9a–d**, respectively, in good yields (Schemes 1 and 2).

The structures of the reaction products **5a–d** and **9a–d** were established and confirmed by their elemental analysis and spectral data (MS, IR, ^1^HNMR, ^13^CNMR). Thus, the structure of **5a** is supported by its mass spectrum, which showed a molecular ion corresponding to the formula C_23_H_15_N_3_O_3_ (M^+^, 381). The ^1^H-NMR spectrum showed characteristic signals at *δ *= 2.41 (s, 3H, CH_3_), 7.02–7.88 (m, 9H, Ar–H), 7.96 (d, 1H, *J* = 4.8 Hz, pyridine-H5), 8.33 (d, 1H, *J* = 4.8 Hz, pyridine-H4), 9.22 (s, 1H, Coumarin-H4) ppm. Its IR spectrum showed the characteristic bands at *v *= 2226 (CN), 1725, 1673 (2C=O) cm^−1^.

To account for the formation of the products **5a–d** and **9a–d**, it is suggested that the studied reactions started with a nucleophilic attack by the methylene group of compound **3** at the formyl group of compound **2** or **7**, which formed in situ due to the reaction of the formyl salts with water. This resulted in the formation of the non-isolable intermediate **4** or **8**, followed by cyclization through the elimination of the water molecule, leading to the formation of the final pyridine derivatives **5** or **9** (Schemes 1 and 2).

Similarly, the 2-cyano-*N*-(1-substituted ethylidene)acetohydrazides **3a–d** reacted with the appropriate sodium (2‐oxocyclopentylidene)methanolate (**11a**) [32] or sodium 2‐oxocyclohexylidene)methanolate (**11b**) [32] in acetic acid containing piperidine acetate to give 2-oxo-1-((1-aryl(heteryl)ethylidene)amino)-1*H*-cycloalkana[b]pyridine-3-carbonitrile derivatives **13a–h**, respectively (Scheme 3). The structure of **13a–d** has been assigned as a reaction product on the basis of analytical and spectral data. The IR spectrum displayed absorption bands at 2227 cm^−1^ due to C≡N function, at 1670 cm^−1^ due to amidic C=O function. The ^1^H-NMR spectrum (DMSO–*d*_6_) exhibited one singlet signal at *δ* = 2.41 ppm assignable to methyl protons, multiplet signals at *δ* = 1.27–1.85 (m, 8H, 4CH_2_), 2.18–2.26 (m, 1H, CH), 2.41 (s, 3H, CH_3_), 3.42 (m, 1H, CH), 3.75 (m, 1H, CH), in addition to a multiplet signal at *δ* 7.24–7.75 ppm, due to aromatic protons. The mass spectrum showed a molecular ion peak at *m/z *= 281, corresponding to the molecular formula C_17_H_19_N_3_O.

As depicted in Scheme 3, the formation of **10** seems to start with an initial attack by a carbanion of the active methylene compound **3** to the formyl group of the salt **11,** which formed in situ due to the reaction of the formyl salts **11** with water, forming. Subsequent enolization followed by elimination of water led to product **13**.

### Antitumor activity

The antitumor activity of compounds **5a–d, 9a–d** and **13a–d** was determined against a liver carcinoma cell line, HEPG2. Doxorubicin was utilized as a reference drug and showed IC50 = 0.72 μM against this liver carcinoma cell line. Collected data were used to plot a dose–response curve, of which the concentration (μM) of the tested compounds required to kill of 50% of the cell population (IC_50_). Antitumor activity was expressed as the mean IC_50_ of three different experiments.

**Fig. 1:**
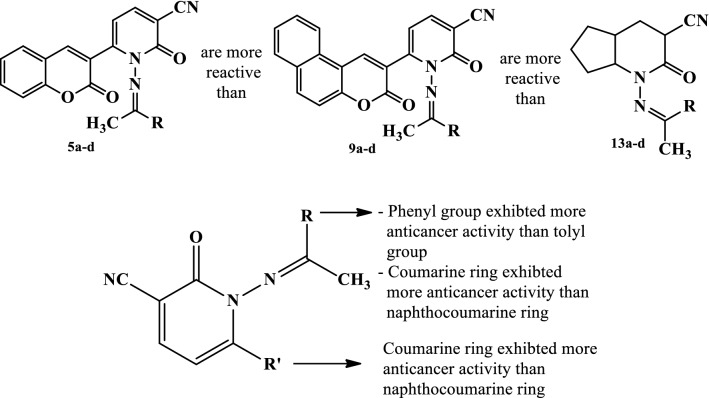
Cytotoxic activities of tested compounds against liver carcinoma cell line

The outcomes showed that the vast majority of the tested compounds demonstrated extraordinary variable activity contrasted with the reference drug, as shown in Table 1 and Fig. 1. The descending order of activity of the new compounds was as follows: **5c > 5d > 5a > 13c > 5b > 9a > 9b > 9d > 13d > 13a > 13b**.

**Table 1:** Cytotoxic activities of tested compounds against liver carcinoma cell line (HEPG2)
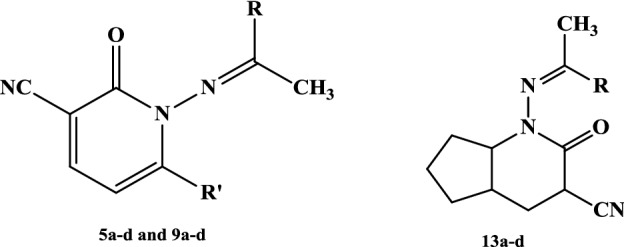

Compd no.	R	R′	IC_50_ (μM)
Doxorubicin	–	–	0.72
**5a**	C_6_H_5_		22.3
**5b**	4-MeC_6_H_4_		40.9
**5c**			*1.46*
**5d**			*7.08*
**9a**	C_6_H_5_		42.8
**9b**	4-MeC_6_H_4_		65.3
**9c**			23.9
**9d**			66.5
**13a**	C_6_H_5_	–	74.3
**13b**	4-MeC_6_H_4_		92.5
**13c**			39.0
**13d**			72.4

The most active compounds are in italic

Examination of the SAR leads to the following conclusions.

The pyridine derivatives **5c** and **5d** (IC_50_ = 1.46, 7.08 µM, respectively) demonstrated potent antitumor activity against HEPG2, while pyridines **5a**, **9c**, **13c**, **5b**, **9a**, showed moderate activity (IC_50_ = 22.3–42.8 µM). The remaining pyridines showed poor antitumor activity against this liver carcinoma cell line (IC_50_ > 65 µM).

The pyridine derivatives having coumarine ring **5a–d** exhibited more anticancer activity than pyridines having naphthocoumarine ring **9a–d** while the latter pyridines **9a–d** exhibited more activity than cyclopenta[b]pyridines **13a–d.**

### Experimental section

Melting points were recorded in open capillaries using an electrothermal Gallenkamp apparatus and are uncorrected. Elemental analyses were carried out by the microanalytical center at Cairo University. The ^1^H and ^13^C NMR spectra were recorded in DMSO-*d*_6_ on a Bruker DRX NMR spectrometer operating at 400 MHz for ^1^H and 100 MHz for ^13^C NMR. Chemical shift (*δ*) values are expressed in ppm and are referenced to the residual solvent signals of DMSO-*d*_6_. The mass spectra were recorded on GCMSQ1000-EX Shimadzu spectrometers. The IR spectra were measured on a Pye-Unicam SP300 instrument.

### Synthesis of the sodium salt of 3-(3-hydroxyprop-2-enoyl)-2*H*-chromen-2-one (4) and the sodium salt of 2-(-3-hydroxyprop-2-enoyl)-3*H*-benzo[f]chromen-3-one (7)

Sodium methoxide (0.054 g, 10 mmol) and ether (20 mL) were poured through a separating funnel to a three-necked flask (250 mL), then the appropriate 3-acetyl-2*H*-chromen-2-one (**1**) or 2-acetyl-3*H*-benzo[*f*]chromen-3-one (**6**) (10 mmol of each) and ethyl formate (0.74 g, 10 mmol) were added and stirred. The formed solid products **4** and **7** were collected via filtration and used directly in the following reactions.

### Synthesis of 2-oxo-1,2-dihydropyridine-3-carbonitrile derivatives 5a–d and 9a–d

An aqueous solution of **4** or **7** (10 mmol of each), the appropriate cyanoacetic acid hydrazones **3a–d** (10 mmol) and piperidine acetate (1 mL) was refluxed for 10 min, then acetic acid (1.5 mL) was added to the hot solution. The formed product was separated and recrystallized from the suitable solvent to yield products **5a–d** or **9a–d.** The analytical data of the obtained products **5a–d** and **9a–d** are listed below:

#### 2-Oxo-6-(2-oxo-2*H*-chromen-3-yl)-1-((1-phenylethylidene)amino)-1,2-dihydropyridine-3-carbonitrile (5a)

Yield 81%; yellow solid; mp 182–184 °C (EtOH); IR (KBr): *v* 3038, 2926 (C–H), 2226 (CN), 1725, 1673 (2C=O), 1603 (C=N) cm^−1^; ^1^H NMR (DMSO-*d*_6_): *δ* 2.41 (s, 3H, CH_3_), 7.02–7.88 (m, 9H, Ar–H), 7.96 (d, 1H, *J* = 4.8 Hz, pyridine-H5), 8.33 (d, 1H, *J* = 4.8 Hz, pyridine-H4), 9.22 (s, 1H, Coumarin-H4) ppm; ^13^C NMR (DMSO-*d*_6_): *δ* 18.3 (CH_3_), 96.7, 116.3, 118.5, 120.3, 123.6, 126.2, 127.6, 127.9, 128.4, 129.0, 129.6, 132.6, 134.3, 134.8, 142.5, 147.1, 156.4, 158.0.2 (Ar–C), 162.4, 163.1 (2C=O) ppm; MS *m/z* (%): 381 (M^+^, 25), 352 (69), 203 (81), 104 (85), 64 (100). Anal. Calcd for C_23_H_15_N_3_O_3_ (381.38): C, 72.43; H, 3.96; N, 11.02. Found C, 72.31; H, 3.89; N, 10.96.

#### 2-Oxo-6-(3-oxo-3*H*-benzo[f]chromen-2-yl)-1-[(1-phenylethylidene)amino]-1,2-dihydropyridine-3-carbonitrile (5b)

Yield 83%; yellow solid; mp 206–208 °C (EtOH); IR (KBr): *v* 3027, 2929 (C–H), 2227 (CN), 1727, 1673 (2C=O), 1601 (C=N) cm^−1^; ^1^H NMR (DMSO-*d*_6_): *δ* 2.31 (s, 3H, CH_3_), 2.42 (s, 3H, CH_3_), 7.13–7.80 (m, 8H, Ar–H), 7.90 (d, 1H, *J* = 4.8 Hz, pyridine-H5), 8.26 (d, 1H, *J* = 4.8 Hz, pyridine-H4), 9.12 (s, 1H, Coumarin-H4) ppm; ^13^C NMR (DMSO-*d*_6_): *δ* 18.0, 22.4 (CH_3_), 94.7, 117.5, 119.3, 120.2, 121.9, 124.8, 125.0, 127.3, 127.7, 128.8, 129.1, 130.2, 132.6, 133.8, 140.2, 149.2, 155.1, 157.9 (Ar–C), 162.0, 164.2 (2C=O) ppm; MS *m/z* (%): 395 (M^+^, 18), 315 (37), 203 (58), 91 (80), 64 (100). Anal. Calcd for C_24_H_17_N_3_O_3_ (395.41): C, 72.90; H, 4.33; N, 10.63. Found C, 72.98; H, 4.27; N, 10.51.

#### 2-Oxo-6-(2-oxo-2*H*-chromen-3-yl)-1-((1-(2-oxo-2*H*-chromen-3-yl)ethylidene)amino)-1,2-dihydropyridine-3-carbonitrile (5c)

Yield 78%; yellow solid; mp 231–233 °C (DMF); IR (KBr): *v* 3044, 2936 (C–H), 2229 (CN), 1733, 1728, 1677 (3C=O), 1603 (C=N) cm^−1^; ^1^H NMR (DMSO-*d*_6_): *δ* 2.40 (s, 3H, CH_3_), 7.39–7.93 (m, 8H, Ar–H), 7.92 (d, 1H, *J* = 4.8 Hz, pyridine-H5), 8.13 (d, 1H, *J* = 4.8 Hz, pyridine-H4), 9.12, 9.23 (2 s, 2H, 2Coumarin-H4) ppm; MS *m/z* (%): 449 (M^+^, 52), 362 (47), 250 (61), 144 (85), 91 (100), 64 (79). Anal. Calcd for C_26_H_15_N_3_O_5_ (449.41): C, 69.49; H, 3.36; N, 9.35. Found C, 69.31; H, 3.17; N, 9.19.

#### 2-Oxo-6-(2-oxo-2*H*-chromen-3-yl)-1-((1-(3-oxo-3*H*-benzo[f]chromen-2-yl)ethylidene) amino)-1,2-dihydropyridine-3-carbonitrile (5d)

Yield 80%; yellow solid; mp 244–246 °C (DMF); IR (KBr): *v* 3047, 2922 (C–H), 2221 (CN), 1729, 1718, 1670 (3C=O), 1599 (C=N) cm^−1^; ^1^H NMR (DMSO-*d*_6_): *δ* 2.37 (s, 3H, CH_3_), 7.16–7.88 (m, 10H, Ar–H), 7.92 (d, 1H, *J* = 4.8 Hz, pyridine-H5), 8.26 (d, 1H, *J* = 4.8 Hz, pyridine-H4), 8.93 (s, 1H, Naphthocoumarin-H4), 9.25 (s, 1H, Coumarin-H4) ppm; MS *m/z* (%): 499 (M^+^, 14), 382 (39), 218 (100), 173 (70), 91 (67), 64 (58). Anal. Calcd for C_30_H_17_N_3_O_5_ (499.47): C, 72.14; H, 3.43; N, 8.41. Found C, 72.03; H, 3.26; N, 8.28.

#### 2-Oxo-6-(3-oxo-3*H*-benzo[f]chromen-2-yl)-1-((1-phenylethylidene)amino)-1,2-dihydropyridine-3-carbonitrile (9a)

Yield 77%; yellow solid; mp 206–208 °C (DMF); IR (KBr): *v* 3051, 2929 (C–H), 2226 (CN), 1723, 1670 (2C=O), 1602 (C=N) cm^−1^; ^1^H NMR (DMSO-*d*_6_): *δ* 2.40 (s, 3H, CH_3_), 7.25–7.81 (m, 11H, Ar–H), 7.96 (d, 1H, *J* = 4.8 Hz, pyridine-H5), 8.28 (d, 1H, *J* = 4.8 Hz, pyridine-H4), 8.93 (s, 1H, Naphthocoumarin-H4) ppm, ^13^C NMR (DMSO-*d*_6_): *δ* 18.8 (CH_3_), 95.8, 103.7, 116.7, 119.5, 121.0, 122.7, 123.7, 126.1, 127.2, 127.7, 128.0, 128.6, 129.1, 130.4, 131.4, 132.6, 134.0, 134.5, 145.9, 155.3, 157.6 (Ar–C), 162.1, 164.3 (2C=O) ppm; MS *m/z* (%): 431 (M^+^, 36), 306 (58), 218 (36), 139 (42), 91 (77), 64 (100). Anal. Calcd for C_27_H_17_N_3_O_3_ (431.44): C, 75.16; H, 3.97; N, 9.74. Found C, 75.03; H, 3.91; N, 9.59.

#### 2-Oxo-6-(3-oxo-3*H*-benzo[f]chromen-2-yl)-1-((1-(*p*-tolyl)ethylidene)amino)-1,2-dihydropyridine-3-carbonitrile (9b)

Yield 82%; yellow solid; mp 222–224 °C (DMF); IR (KBr): *v* 3041, 2935 (C–H), 2218 (CN), 1733, 1682 (2C=O), 1601 (C=N) cm^−1^; ^1^H NMR (DMSO-*d*_6_): *δ* 2.30 (s, 3H, CH_3_), 2.43 (s, 3H, CH_3_), 7.17–7.81 (m, 10H, Ar–H), 7.95 (d, 1H, *J* = 4.8 Hz, pyridine-H5), 8.41 (d, 1H, *J* = 4.8 Hz, pyridine-H4), 8.94 (s, 1H, Naphthocoumarin-H4) ppm; ^13^C NMR (DMSO-*d*_6_): *δ* 18.4, 22.7 (CH_3_), 94.7, 105.9, 117.0, 120.4, 120.9, 121.4, 122.0, 124.8, 126.3, 127.0, 128.7, 128.6, 129.8, 131.4, 131.8, 133.6, 135.3, 136.0, 142.6, 151.4, 155.3 (Ar–C), 163.6, 165.1 (2C=O) ppm; MS *m/z* (%): 445 (M^+^, 100), 341 (36), 265 (54), 182 (74), 64 (83). Anal. Calcd for C_28_H_19_N_3_O_3_ (445.47): C, 75.49; H, 4.30; N, 9.43. Found C, 75.32; H, 4.16; N, 9.27.

#### 2-Oxo-1-((1-(2-oxo-2*H*-chromen-3-yl)ethylidene)amino)-6-(3-oxo-3*H*-benzo[f]chromen-2-yl)-1,2-dihydropyridine-3-carbonitrile (9c)

Yield 80%; brown solid; mp 231–233 °C (DMF); IR (KBr): *v* 3040, 2961 (C–H), 2223 (CN), 1739, 1726, 1675 (3C=O), 1597 (C=N) cm^−1^; ^1^H NMR (DMSO-*d*_6_): *δ* 2.41 (s, 3H, CH_3_), 7.27–7.93 (m, 10H, Ar–H), 8.03 (d, 1H, *J* = 4.8 Hz, pyridine-H5), 8.38 (d, 1H, *J* = 4.8 Hz, pyridine-H4), 8.97 (s, 1H, Naphthocoumarin-H4), 9.13 (s, 1H, Coumarin-H4) ppm; MS *m/z* (%): 499 (M^+^, 36), 360 (51), 218 (100), 154 (73), 104 (55), 64 (81). Anal. Calcd for C_30_H_17_N_3_O_5_ (499.47): C, 72.14; H, 3.43; N, 8.41. Found C, 72.01; H, 3.25; N, 8.27.

#### 2-Oxo-6-(3-oxo-3*H*-benzo[f]chromen-2-yl)-1-((1-(3-oxo-3*H*-benzo[f]chromen-2-yl)ethylidene)amino)-1,2-dihydropyridine-3-carbonitrile (9d)

Yield 76%; brown solid; mp 271–273 °C (DMF); IR (KBr): *v* 3042, 2938 (C–H), 2229 (CN), 1736, 1729, 1676 (3C=O), 1607 (C=N) cm^−1^; ^1^H NMR (DMSO-*d*_6_): *δ* 2.44 (s, 3H, CH_3_), 7.41–7.95 (m, 12H, Ar–H), 8.12 (d, 1H, *J* = 4.8 Hz, pyridine-H5), 8.46 (d, 1H, *J* = 4.8 Hz, pyridine-H4), 8.89, 8.93 (2 s, 2H, 2Naphthocoumarin-H4) ppm: MS *m/z* (%): 549 (M^+^, 22), 315 (62), 288 (67), 154 (100), 91 (38), 64 (77). Anal. Calcd for C_34_H_19_N_3_O_5_ (549.53): C, 74.31; H, 3.48; N, 7.65. Found C, 74.18; H, 3.29; N, 7.44.

### Synthesis of sodium salt of cycloalkanones 11a, b

In a three-necked flask (250 mL), sodium methoxide (0.054 g, 10 mmol) and ether (20 mL) were poured through a separating funnel, the appropriate cyclopentanone (**10a**) or cyclohexanone (**10b**) (10 mmol of each) with ethyl formate (0.74 g, 10 mmol) were added, and then stirred. The formed solid products **11a** and **11b** were collected and used directly in the following reactions.

### Synthesis of 2-oxo-1,2-dihydropyridine-3-carbonitrile derivatives 13a–h

A solution of **11a** or **11b** (10 mmol of each), the appropriate cyanoacid hydrazones **3a–d** (10 mmol) and piperidine acetate (1 mL) in water (3 mL) was refluxed for 10 min. Acetic acid (1.5 mL) was added to the hot solution. The solid product was filtered off and recrystallized from the proper solvent to give products **13a–h.** The physical constants and spectral data of the obtained products **13a–h** are listed below:

#### 2-Oxo-1-((1-phenylethylidene)amino)octahydro-1*H*-cyclopenta[b]pyridine-3-carbonitrile (13a)

Yield 73%; yellow solid; mp 204–206 °C (EtOH); IR (KBr): *v* 3033, 2925 (C–H), 2227 (CN), 1670 (C=O), 1607 (C=N) cm^−1^; ^1^H NMR (DMSO-*d*_6_): *δ* 1.27–1.85 (m, 8H, 4CH_2_), 2.18–2.26 (m, 1H, CH), 2.41 (s, 3H, CH_3_), 3.42 (m, 1H, CH), 3.75 (m, 1H, CH), 7.24–7.75 (m, 5H, ArH) ppm; ^13^C NMR (DMSO-*d*_6_): *δ* 23.7, 25.3, 28.5, 33.4, 34.9, 36.3, 41.1, 62.0, 95.8, 125.3, 126.2, 129.1, 134.4, 160.3, 171.6 ppm; MS *m/z* (%): 281 (M^+^, 16), 203 (40), 127 (100), 91 (48), 64 (52). Anal. Calcd for C_17_H_19_N_3_O (281.35): C, 72.57; H, 6.81; N, 14.94. Found C, 72.42; H, 6.69; N, 14.70.

#### 2-Oxo-1-((1-(*p*-tolyl)ethylidene)amino)octahydro-1*H*-cyclopenta[b]pyridine-3-carbonitrile **(13b)**

Yield 75%; yellow solid; mp 193–195 °C (EtOH); IR (KBr): *v* 3027, 2948 (C–H), 2221 (CN), 1675 (C=O), 1602 (C=N) cm^−1^; ^1^H NMR (DMSO-*d*_6_): *δ* 1.23–1.87 (m, 8H, 4CH_2_), 2.09–2.18 (m, 1H, CH), 2.31 (s, 3H, CH_3_), 2.43 (s, 3H, CH_3_), 3.39 (m, 1H, CH), 3.72 (m, 1H, CH), 7.36 (d, *J* = 8.1 Hz, 2H, ArH), 7.69 (d, *J* = 8.1 Hz, 2H, ArH) ppm; MS *m/z* (%): 295 (M^+^, 28), 229 (36), 174 (72), 91 (100), 64 (83). Anal. Calcd for C_18_H_21_N_3_O (295.38): C, 73.19; H, 7.17; N, 14.23. Found C, 73.03; H, 7.06; N, 14.03.

#### 2-Oxo-1-((1-(2-oxo-2*H*-chromen-3-yl)ethylidene)amino)octahydro-1*H*-cyclopenta[b] pyridine-3-carbonitrile (13c)

Yield 77%; brown solid; mp 247–249 °C (DMF); IR (KBr): *v* 3051, 2922 (C–H), 2232 (CN), 1728, 1670 (2C=O), 1601 (C=N) cm^−1^; ^1^H NMR (DMSO-*d*_6_): *δ* 1.29–1.88 (m, 8H, 4CH_2_), 2.07–2.15 (m, 1H, CH), 2.37 (s, 3H, CH_3_), 3.46 (m, 1H, CH), 3.79 (m, 1H, CH), 7.52–7.86 (m, 4H, ArH), 8.75 (s, 1H, Coumarin-H4) ppm; MS *m/z* (%): 349 (M^+^, 35), 262 (37), 183 (100), 91 (85), 64 (60). Anal. Calcd for C_20_H_19_N_3_O_3_ (349.38): C, 68.75; H, 5.48; N, 12.03. Found C, 68.83; H, 5.35; N, 11.84.

#### 2-Oxo-1-((1-(3-oxo-3*H*-benzo[f]chromen-2-yl)ethylidene)amino)octahydro-1*H*-cyclopenta[b]pyridine-3-carbonitrile (13d)

Yield 74%; brown solid; mp 260–262 °C (DMF); IR (KBr): *v* 3039, 2938 (C–H), 2230 (CN), 1722, 1668 (2C=O), 1604 (C=N) cm^−1^; ^1^H NMR (DMSO-*d*_6_): *δ* 1.32–1.90 (m, 8H, 4CH_2_), 2.13–2.19 (m, 1H, CH), 2.41 (s, 3H, CH_3_), 3.48 (m, 1H, CH), 3.73 (m, 1H, CH), 7.36–7.88 (m, 6H, ArH), 8.56 (s, 1H, Naphthocoumarin-H4) ppm; MS *m/z* (%): 399 (M^+^, 12), 291 (60), 183 (80), 91 (49), 64 (100). Anal. Calcd for C_24_H_21_N_3_O_3_ (399.44): C, 72.16; H, 5.30; N, 10.52. Found C, 72.03; H, 5.19; N, 10.33.

#### 2-Oxo-1-((1-phenylethylidene)amino)decahydroquinoline-3-carbonitrile (13e)

Yield 73%; yellow solid; mp 204–206 °C (EtOH); IR (KBr): *v* 3033, 2925 (C–H), 2227 (CN), 1670 (C=O), 1607 (C=N) cm^−1^; ^1^H NMR (DMSO-*d*_6_): *δ* 1.18–1.96 (m, 10H, 5CH_2_), 2.04–2.09 (m, 1H, CH), 2.42 (s, 3H, CH_3_), 3.49 (m, 1H, CH), 3.68 (m, 1H, CH), 7.28–7.80 (m, 5H, ArH) ppm; MS *m/z* (%): 295 (M^+^, 100), 239 (43), 160 (73), 91 (63), 64 (48). Anal. Calcd for C_18_H_21_N_3_O (295.38): C, 73.19; H, 7.17; N, 14.23. Found C, 73.10; H, 7.05; N, 14.05.

#### 2-Oxo-1-((1-(*p*-tolyl)ethylidene)amino)decahydroquinoline-3-carbonitrile (13f)

Yield 75%; yellow solid; mp 218–220 °C (EtOH); IR (KBr): *v* 3053, 2940 (C–H), 2231 (CN), 1670 (C=O), 1601 (C=N) cm^−1^; ^1^H NMR (DMSO-*d*_6_): *δ* 1.18–1.96 (m, 10H, 5CH_2_), 2.04–2.09 (m, 1H, CH), 2.42 (s, 3H, CH_3_), 3.49 (m, 1H, CH), 3.68 (m, 1H, CH), 7.31 (d, *J* = 8.1 Hz, 2H, ArH), 7.74 (d, *J* = 8.1 Hz, 2H, ArH) ppm; MS *m/z* (%): 309 (M^+^, 48), 220 (81), 176 (46), 91 (100), 64 (49). Anal. Calcd for C_19_H_23_N_3_O (309.41): C, 73.76; H, 7.49; N, 13.58. Found C, 73.58; H, 7.40; N, 13.42.

#### 2-Oxo-1-((1-(2-oxo-2*H*-chromen-3-yl)ethylidene)amino)decahydroquinoline-3-carbonitrile (13g)

Yield 71%; brown solid; mp 237–239 °C (DMF); IR (KBr): *v* 3041, 29428 (C–H), 2229 (CN), 1727, 1673 (2C=O), 1599 (C=N) cm^−1^; ^1^H NMR (DMSO-*d*_6_): *δ* 1.20–1.92 (m, 10H, 5CH_2_), 2.05–2.15 (m, 1H, CH), 2.42 (s, 3H, CH_3_), 3.43 (m, 1H, CH), 3.76 (m, 1H, CH), 7.44–7.82 (m, 4H, ArH), 8.73 (s, 1H, Coumarin-H4) ppm; MS *m/z* (%): 363 (M^+^, 100), 259 (53), 160 (52), 91 (84), 64 (75). Anal. Calcd for C_21_H_21_N_3_O_3_ (363.41): C, 69.41; H, 5.82; N, 11.56. Found C, 69.57; H, 5.68; N, 11.44.

#### 2-Oxo-1-((1-(3-oxo-3*H*-benzo[f]chromen-2-yl)ethylidene)amino)decahydroquinoline-3-carbonitrile (13h)

Yield 76%; brown solid; mp 263–265 °C (DMF); IR (KBr): *v* 3032, 2928 (C–H), 2224 (CN), 1723, 1675 (2C=O), 1597 (C=N) cm^−1^; ^1^H NMR (DMSO-*d*_6_): *δ* 1.24–1.90 (m, 10H, 5CH_2_), 2.07–2.12 (m, 1H, CH), 2.40 (s, 3H, CH_3_), 3.46 (m, 1H, CH), 3.80 (m, 1H, CH), 7.49–7.77 (m, 6H, ArH), 8.62 (s, 1H, Naphthocoumarin-H4) ppm; MS *m/z* (%): 413 (M^+^, 52), 226 (37), 117 (64), 91 (69), 64 (100). Anal. Calcd for C_25_H_23_N_3_O_3_ (413.47): C, 72.62; H, 5.61; N, 10.16. Found C, 72.46; H, 5.48; N, 10.04.

### Evaluation of the antitumor activity using Viability assay

The cytotoxic evaluation of the synthesized compounds was carried out at the Regional Center for Mycology and Biotechnology at Al-Azhar University, Cairo, Egypt, according to a reported method [33].

## Materials and methods

### Chemicals

All chemicals used in this study are of high analytical grade. They were obtained from (either Sigma-Alderich or Biorad).

### Human tumor cell lines

The tumour cell lines were obtained frozen in liquid nitrogen (− 180 °C) from the American Type Culture Collection (ATCC^®^ HB-8065™) and was maintained at the National Cancer Institute, Cairo, Egypt, by serial sub-culturing.

### Measurement of potential cytotoxic activity

The cytotoxic activity was measured in vitro on human cancer cell line (HEPG2) using Sulforhodamine-B stain (SRB) assay.

Cells were plated in 96 multi well plates for 24 h before treatment with the compounds to allow attachment of the cells to the wall of the plate.

- Different concentrations of the compound under test (0, 6.25, 12.5, 25, 50 and 100 µg/mL) were added to the cell monolayer. Triplicate wells were prepared for each individual dose.
- Monolayer cells were incubated with the compounds for 48 h at 37 °C and in atmosphere of 5% CO_2_.
- After 48 h cell was fixed, washed and stained with Sulforhodamine B stain.
- The relation between surviving fraction and drug concentration was plotted and IC_50_ (the concentration required for 50% inhibition of cell viability) was calculated for each compound by Sigma-plot software.

## Conclusions

The results of the present study indicate that the cyanoacid hydrazones and sodium 3-oxo-3-heterylprop-1-en-1-olates or sodium (2-oxocycloalkylidene)methanolates are useful precursors for the synthesis of various functionalized 3-pyridinecarbonitriles. In addition, they indicate that these reactions are region-specific, as in each case, one product of good yield was produced. Most of the synthesized compounds were evaluated for their anti-cancer activity against the liver carcinoma cell lines. Also, their structure activity relationship (SAR) was studied. The results revealed that the pyridine derivatives **5c** and **5d** (IC_50_ = 1.46, 7.08 µM, respectively) have promising antitumor activity against liver carcinoma cell line (HEPG2). The prepared compounds are expected to be of pharmacological interest.

## Data Availability

The datasets and samples of the compounds used during the current study are available from the corresponding author on reasonable request.

## Abbreviations

TLC: thin layer chromatography
HepG2: liver carcinoma cell line
EtOH: ethanol
DMF: dimethylformamide
m.p.: melting point
IR: infra- red
ATCC: American Type Culture Collection
SAR: structure activity relationship
